# Clinical and endocrine characteristics and genetic analysis of Korean children with McCune–Albright syndrome: a retrospective cohort study

**DOI:** 10.1186/s13023-016-0496-x

**Published:** 2016-08-09

**Authors:** Eun-Kyung Cho, Jinsup Kim, Aram Yang, Chang-Seok Ki, Ji-Eun Lee, Sung Yoon Cho, Dong-Kyu Jin

**Affiliations:** 1 Department of Pediatrics, Samsung Medical Center, Sungkyunkwan University School of Medicine, 81 Irwon-ro, Gangnam-gu, Seoul 06351 Republic of Korea; 2 Department of Laboratory Medicine & Genetics, Samsung Medical Center, Sungkyunkwan University School of Medicine, Seoul, Republic of Korea; 3 Department of Pediatrics, Inha University Hospital, Inha University Graduate School of Medicine, Incheon, Republic of Korea

**Keywords:** McCune–Albright syndrome, *GNAS*, Peripheral precocious puberty, Fibrous dysplasia, Pituitary adenoma, Growth hormone excess, MEMO-PCR

## Abstract

**Background:**

McCune–Albright syndrome (MAS) is a rare disease defined by the triad of fibrous dysplasia (FD), café au lait spots, and peripheral precocious puberty (PP). Because of the rarity of this disease, only a few individuals with MAS have been reported in Korea. We describe the various clinical and endocrine manifestations and genetic analysis of 14 patients with MAS in Korea.

**Methods:**

Patients’ clinical data—including peripheral PP, FD, and other endocrine problems—were reviewed retrospectively. In addition, treatment experiences of letrozole in five patients with peripheral PP were described. Mutant enrichment with 3′-modified oligonucleotides - polymerase chain reaction (MEMO-PCR) was performed on eight patients to detect mutation in *GNAS* using blood. MEMO-PCR is a simple and practical method that enables the nondestructive selection and enrichment of minor mutant alleles in blood.

**Results:**

The median age at diagnosis was 5 years 2 months (range: 18 months to 16 years). Eleven patients were female, and three were male. Thirteen patients showed FD. All female patients showed peripheral PP at onset, and three patients subsequently developed central PP. There was a significant decrease in estradiol levels after two years of letrozole treatment. However, bone age was advanced in four patients. Two patients had clinical hyperthyroidism, and two patients had growth hormone (GH) excess with pituitary microadenoma. c.602G > A (p.Arg201His) in *GNAS* was detected in two patients in blood, and c.601C > T (p.Arg201Cys) in *GNAS* was detected in one patient in pituitary adenoma.

**Conclusions:**

This study described the various clinical manifestations of 14 patients with MAS in a single center in Korea. This study first applied MEMO-PCR on MAS patients to detect *GNAS* mutation. Because a broad spectrum of endocrine manifestations could be found in MAS, multiple endocrinopathies should be monitored in MAS patients. Better treatment options for peripheral PP with MAS are needed.

## Background

McCune–Albright syndrome (MAS) is a rare congenital sporadic disorder, and the precise prevalence of MAS is not known (the estimated prevalence ranges between 1/100,000 and 1/1,000,000) [1]. MAS is defined by the triad of polyostotic fibrous dysplasia of bone (FD), café au lait skin pigmentation, and peripheral precocious puberty (PP). Other multiple endocrinopathies—including hyperthyroidism, growth hormone (GH) excess, hypercortisolism, and renal phosphate wasting—could be associated with the original triad [2].

Peripheral PP is the most common endocrine manifestation of MAS, and it is much more frequently found in girls than in boys [2]. It arises due to the autonomous activation of ovarian tissue [2]. Current treatment of peripheral PP in girls with MAS revolves around the use of anti-estrogens, including aromatase inhibitors (AIs) and estrogen receptor modulators [3]. We tried to evaluate the efficacy and safety of letrozole, a third-generation AI, in girls with peripheral PP-associated MAS.

This syndrome is caused by a postzygotic somatic activating mutation in the *GNAS* gene encoding the G-protein alpha subunit (Gsα). Activating Gsα mutations that induce constitutive activation of the cAMP signaling pathway leads to multiple clinical manifestations [4]. In MAS, mutations are exclusively present in the somatic mosaic state, and mutation abundance is generally low in unaffected tissues. Thus, it is difficult to detect mutations in peripheral blood leukocytes by standard Sanger sequencing. However, biopsy of affected tissue to identify the genetic defect is too invasive, requiring surgical intervention. In this regard, we applied the mutant enrichment with 3′-modified oligonucleotides - polymerase chain reaction (MEMO-PCR) method for the detection of even low levels of mutant alleles using peripheral blood leukocytes.

Because of the rarity of this disease, only a few patients with MAS have been reported in Korea. Here, we describe the various clinical manifestations and genetic analysis of 14 patients with MAS in a single center in Korea.

## Methods

We performed a retrospective study on 14 patients with MAS who were followed over 16 years (1999–2015) at the Samsung Medical Center. The diagnoses were made based on the following clinical criteria. Patients were required to exhibit at least two of the three major features of MAS (hyperfunctioning endocrinopathies, polyostotic FD, and café au lait spots) [1]. Initial evaluation of MAS included laboratory and radiographic studies (skeletal surveys). Eight patients underwent genetic studies of peripheral blood or affected tissue. Written informed consents were obtained from the parents of each patient, and the Institutional Review Board approved the study (IRB file number: 2012-12-054).

### Endocrinopathies

Eleven girls with clinically suspected PP were firstly evaluated for serum levels of luteinizing hormone (LH), follicle-stimulating hormone (FSH), and estradiol at baseline. A gonadotropin-releasing hormone (GnRH) stimulation test was then performed to differentiate gonadotropin-dependent PP from gonadotropin-independent PP. X-rays of the hand and wrist to determine bone age were checked regularly for patients diagnosed with PP. Patients with vaginal bleeding were questioned about episodes of menstruation at every follow-up. In addition, patients were assessed with pelvic ultrasound for measurement of uterine and ovarian volumes and evaluation of abnormal findings, such as ovarian cysts.

In addition, we evaluated thyroid-stimulating hormone (TSH), total triiodothyronine (T3), and free thyroxine (FT4). A GH suppression test was performed for two patients (Patients 3 and 12) with tall stature and acromegalic features, and a brain MRI was done for these patients to localize the source of GH excess.

### Fibrous dysplasia

The diagnosis of FD was established on a clinical and radiological basis for all patients and from bone biopsy for five patients who underwent orthopedic surgery due to pathological fracture. Plain radiographs are often sufficient to diagnose FD. Eight patients had a bone scan to determine the extent of the disease. Among the patients clinically suspected of craniofacial FD, craniofacial computed tomography (CT) was performed in eight.

### Genetic analysis

Eight out of the 14 patients agreed to perform genetic tests for MAS. After obtaining informed consent, genomic DNA was extracted from peripheral blood leukocytes using the Wizard Genomic DNA Purification kit following the manufacturer’s instructions (Promega, Madison, WI). The exon 8 region of the *GNAS* gene was tested by conventional Sanger sequencing with a primer set (forward: 5′-ggactctgagccctctttcc-3′, reverse: 5′-accacgaagatgatggcagt-3′) as well as MEMO-PCR using a primer set (forward: 5′-tgtttcaggacctgcttcg-3′, reverse: 5′-gaacagccaagcccacag-3′, blocking: 5′-cttcgctgccgtgtcctg-6-amine-3′) followed by sequencing with the reverse primer. The PCR was performed with a thermal cycler (model Veriti, Applied Biosystems, Foster City, CA, USA), and sequencing was performed with the BigDye Terminator Cycle Sequencing Ready Reaction kit (Applied Biosystems) on the ABI Prism 3100xl genetic analyzer (Applied Biosystems). To describe sequence variations, we followed the guidelines by the Human Genome Nomenclature Committee (HGVS) such that “A” of the ATG translation start site was numbered +1 for a DNA sequence and the first methionine was numbered +1 for a protein sequence. We additionally performed Sanger sequencing using brain tissue with pituitary microadenoma on Patient 12.

### Statistical analysis

The mean changes of hormone levels and uterine sizes between before and two years after treatment with letrozole were compared using a paired t test; *p* < 0.05 was considered statistically significant, and data are expressed as means ± standard deviations (SD). The statistical analyses were performed using the SPSS program (version 21.0).

## Results

The median age at diagnosis of MAS was 5 years 2 months (range: 18 months to 16 years). All patients had been diagnosed as having MAS by the time they were aged 16 years or younger, with 12 patients having been diagnosed before 10 years of age. Five patients had been diagnosed by the time they were aged 3 years or younger. The proportion of female patients (79 %) was overwhelmingly higher than that of male patients (21 %). Patients’ clinical characteristics are summarized in Table 1. The most common symptoms at diagnosis were vaginal bleeding or breast development in female patients (7/11, 64 %) and pathological fracture in male patients (2/3, 67 %).

**Table 1:** Clinical manifestations of patients with McCune–Albright syndrome

Patient	Sex	Age at diagnosis (years.months)	Symptoms at diagnosis	SD	FD	PPP	Other endocrinopathies	Genetic analysis
1	F	5	Breast development	+	+	+		NA
2	F	6	Orbital area swelling, left	-	+	+		NA
3	M	5.3	Pathological fracture, right femur	+	+	-	GHH^b^	Detected, blood^c^ (Arg201His)
4	M	1.9	Pathological fracture, left femur	+	+	-	HT, HP	Detected, blood^c^ (Arg201His)
5	F	9	Forehead swelling, left	+	+	+^a^		ND
6	F	11	Vaginal bleeding	+	+	+		ND
7	F	3.4	Vaginal bleeding	+	+	+		NA
8	F	6.7	Vaginal bleeding	-	+	+^a^		ND
9	F	3	Vaginal bleeding	+	+	+		ND
10	F	1.6	Vaginal bleeding	+	+	+	HT, HP	NA
11	F	7.1	Exophthalmos, left	+	+	+^a^		NA
12	M	16	Headache	-	+	-	GHH^b^	Detected, pituitary adenoma (Arg201Cys)
13	F	4	Vaginal bleeding	-	+	+		NA
14	F	3.1	Vaginal bleeding	+	-	+		ND

*SD* skin dysplasia (café au lait spots), *FD* fibrous dysplasia, *PPP* peripheral precocious puberty, *GHH* growth hormone hypersecretion, *HT* hyperthyroidism, *HP* hypophosphatemia, *NA* not available, *ND* not detected

^a^ Patients who subsequently developed central precocious puberty

^b^ Patients exhibited a pituitary adenoma by pituitary MRI

^c^ *GNAS* mutation was detected by MEMO-PCR

### Precocious puberty

Eleven out of 14 patients showed symptoms of peripheral PP. All patients with peripheral PP were female. The median age at onset of initial symptoms of peripheral PP was 3 years (range: 18 months to 6 years 7 months). Peripheral PP was confirmed in 9 out of 11 patients using a GnRH stimulation test, and one (Patient 8) of them subsequently developed central PP during the treatment of peripheral PP. Two patients (Patients 5 and 11) exhibited central PP at diagnosis through a GnRH stimulation test. It was assumed that these two patients had peripheral PP before from the history of vaginal bleeding in early childhood (at the ages of 2 and 3 years old, respectively). Patient 5 was diagnosed as having central PP late at 9 years old; therefore, she was monitored for symptoms of pubertal progression without GnRH analogue treatment. Patient 11 was diagnosed as having central PP at the age of 7 years 1 month and treated with GnRH analogue therapy. Because she showed frequent vaginal bleeding, letrozole treatment was added to GnRH analogue therapy at the age of 8 years. Patient 8 started to receive letrozole treatment at the age of 6 years 7 months but subsequently developed central PP at the age of 8 years and started to receive GnRH analogue therapy.

We analyzed the results of two-year treatment in five patients (Patients 7, 8, 9, 10, and 11) treated with letrozole, the third-generation AI, and followed up regularly in our pediatric endocrinology clinic. Letrozole was initiated orally at a dose of 0.5 mg/m^2^ daily, and the dose was gradually increased up to 1.5–2 mg/m^2^ within a year. Individual results for skeletal maturation, vaginal bleeding, and Tanner staging are shown in Table 2.

**Table 2:** Clinical response to two years of letrozole treatment of McCune–Albright syndrome patients with precocious puberty

		At start of letrozole treatment					Two years after letrozole treatment			
Patient	Sex	CA (years)	Height (cm) (SDS)	BA-CA (months)	Vaginal bleeding	Tanner stage	Height (cm) (SDS)	BA-CA (months)	Vaginal bleeding	Tanner stage
7	F	3.3	96.9 (−0.4)	8	+	IIIB	110 (−0.5)	4	-	IIB
8	F	6.7	132.4 (1.9)	26	+	IIIB	144.4 (1.9)	29	-	IIIB
9	F	6.7	135.6 (2.4)	6	+	IVB	146.3 (2.2)	31	+	IVB
10	F	2.2	86.7 (−1.7)	4	+	IIIB	93.9 (−1.8)	26	+	IIB
11	F	8.1	134.5 (1.4)	11	+	IIIB	143.4 (0.8)	15	+	IIIB

*CA* chronological age, *SDS* standard deviation score, *BA* bone age

All five patients had experiences of vaginal bleeding at diagnosis, and two patients (Patients 7 and 8) showed a reduction in the frequency of menstruation while taking letrozole. No significant changes in the pubertal stages of breasts were seen throughout the study period. Pelvic ultrasound examination revealed ovarian cysts in four patients (Patients 7, 9, 10, and 11) during the treatment periods. Three patients had a unilateral cyst (Patients 7, 10, and 11). The ovarian cyst had disappeared one year after letrozole treatment in Patient 7, and the ovarian cyst size had decreased after two years of treatment in Patient 10. Patient 9 showed bilateral ovarian cysts before treatment, and a left ovarian cyst disappeared during the treatment period. The unilateral ovarian cyst had newly appeared after two years’ treatment in Patient 11. Average uterus lengths had increased from 49.8 ± 6.9 mm to 55.2 ± 18.1 mm (*p* = 0.44), and average widths had also increased from 13 ± 1.9 mm to 16.2 ± 7.9 mm (*p* = 0.41) after letrozole treatment. However, there were no significant differences in uterine size. Hormone levels and pelvic ultrasound findings are shown in Table 3. All five patients experienced a significant decrease in serum estradiol on treatment. After two years’ treatment, average levels of estradiol had decreased from 63.4 ± 40.8 pg/ml to 2.2 ± 1.1 pg/ml (*p* ≤ 0.03). However, LH and FSH levels showed no significant change before and after letrozole treatment. The bone age advancement (defined as bone age – chronological age) was decreased in Patient 7; however, the other four patients showed further advanced bone age. There was no significant change in the height standard deviation score (SDS) during the treatment period. The treatment was well tolerated, and no significant adverse events, such as ovarian torsion, occurred in any patient treated with letrozole.

**Table 3:** Hormone levels and pelvic ultrasonography findings of McCune–Albright patients with precocious puberty

	At start of letrozole treatment						Two years after letrozole treatment				
Patient	LH (mIU/ml)	FSH (mIU/ml)	Estradiol (pg/ml)	Peak LH (mIU/ml)	Ovarian cyst size (mm)	Uterine size^a^ (mm)	LH (mIU/ml)	FSH (mIU/ml)	Estradiol (pg/ml)	Ovarian cyst size (mm)	Uterine size^a^ (mm)
7	0.3	0.3	84	1	Right. 11	50 × 13	0.7	2.4	1	no cyst	36 × 10
8	0.6	0.2	10	3.9	no cyst	57 × 15	1.9	0.4	1	no cyst	63 × 18
9	0.6	1.2	10	1.5	Left. 28						
					Right. 46	45 × 10	1.1	0.3	3	Right. 12	52 × 19
10	0.2	0.1	110	1	Right. 21	41 × 13	1.3	0.2	3	Right. 20	43 × 7
11	1	0.1	10	7.4	no cyst	56 × 14	1.3	0.6	3	Left. 35	82 × 27

*LH* luteinizing hormone, *FSH* follicle-stimulating hormone

^a^ Uterine size is described by length × width

### Hyperthyroidism

Four patients (4/14, 29 %) showed abnormal findings of TSH and/or FT4; three were female (Patients 6, 8, and 10), and one was male (Patient 4). Two patients (Patients 4 and 10) showed increased FT4 levels and confirmed clinical hyperthyroidism (age at diagnosis: 2 and 2.5 years, respectively). Patient 10 presented with tachycardia at diagnosis, and innumerable small cystic lesions in the thyroid gland were revealed by thyroid ultrasound. Medical treatment with methimazole was started, and euthyroid status was achieved in these two patients. In Patients 6 and 8, TSH had decreased, while FT4 levels remained in the normal range. In addition, Patient 8 revealed small cystic nodules in the thyroid gland by thyroid ultrasound. They have been regularly checked up on for TSH and thyroid hormone without treatment.

### Pituitary adenoma producing GH 

GH excess was observed in two patients (Patients 3 and 12). Patient 12 developed acromegaly at the age of 17 years. GH was not suppressed in the GH-suppression test. Pituitary MRI revealed a left pituitary adenoma 7 mm in size without a significant change in FD. A bone scan revealed polyostotic FD in the craniofacial bone and left iliac bone. After tumor removal by endoscopic endonasal surgery, GH was suppressed well. Pituitary pathology revealed a pituitary adenoma. Patient 3 was diagnosed with MAS at 5 years 3 months and initially presented with FD of the craniofacial bones and café au lait spots. During follow-up, this patient showed GH excess at the age of 14 years, and surgery for tumor removal has been planned.

### Renal phosphate wasting

Two patients (Patients 4 and 10) had renal phosphate wasting with hypophosphatemia and received phosphate supplements; neither had signs of rickets on X-ray findings.

### Fibrous dysplasia

Thirteen patients had polyostotic FD. The most common sites of FD involvement were the craniofacial bones. All 13 patients with FD had craniofacial FD, and three patients had FD only in the craniofacial bones. FD in the craniofacial bones and limbs was found in eight patients, and involvement of the axial skeleton was found in two patients (Table 4). FD in the extremities usually presented with a pathological fracture (Patients 3 and 4), and a painless “lump” or asymmetric feature was the presenting sign when FD occurred in the craniofacial bones (Patients 2, 5, and 11).

**Table 4:** Affected sites of fibrous dysplasia

Affected site	No. of patients
Craniofacial	13 (100 %)
Craniofacial only	3 (23 %)
Craniofacial + extremities	8 (62 %)
Craniofacial + extremities + axial	2 (15 %)

### Genetic analysis

**Fig. 1:**
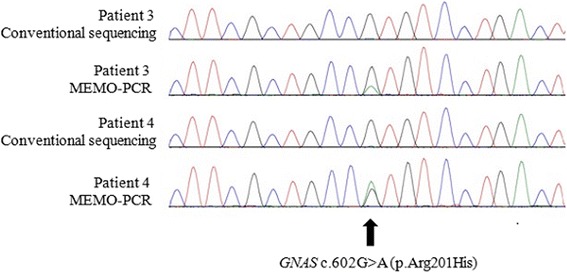
Conventional Sanger sequencing and MEMO-PCR of exon 8 in *GNAS* gene from Patients 3 and 4: MEMO-PCR revealed the Arg201His mutation in both patients from peripheral blood leukocytes. However, conventional Sanger sequencing did not detect *GNAS* mutations

**Fig. 2:**
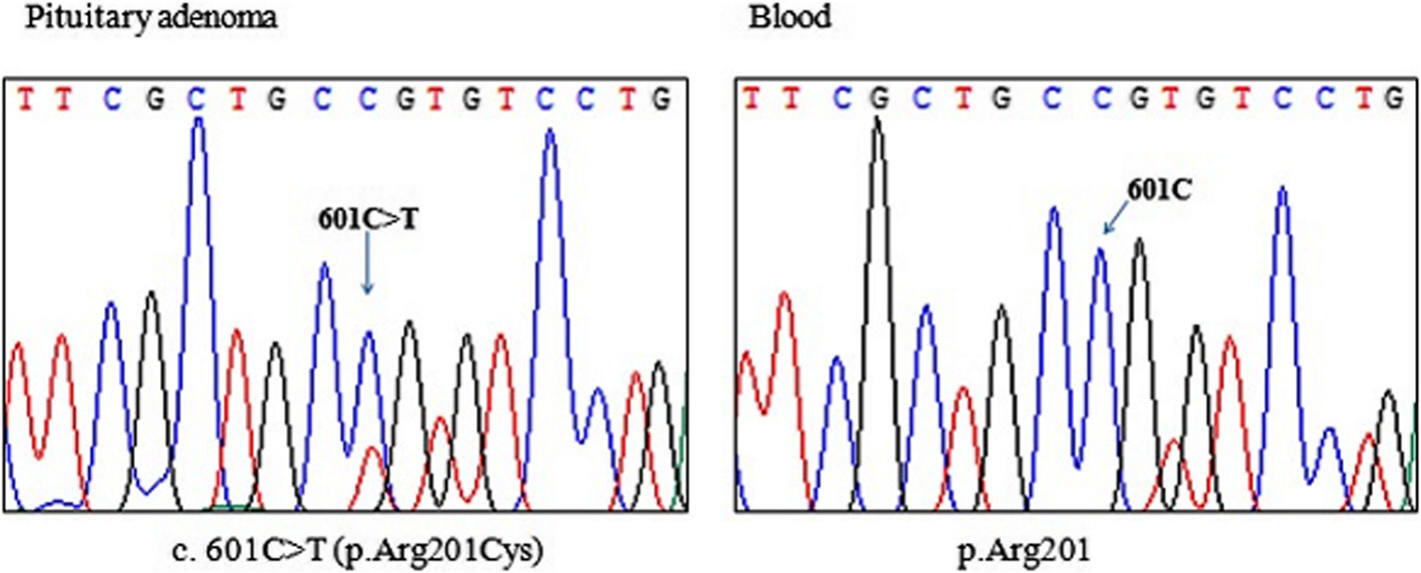
DNA sequencing of *GNAS* gene from Patient 12: Arg201Cys mutation was only detected in pituitary adenoma tissue and not in peripheral blood leukocytes. A heterozygous peak (indicated with arrow) was observed at position 201

Of the eight patients who underwent genetic testing for mutations in *GNAS* in peripheral blood, *GNAS* mutations (p.Arg201His) were detected in two (Patients 3 and 4) by MEMO-PCR (Fig. 1). In the case of Patient 12, who was diagnosed with a pituitary adenoma, *GNAS* mutation (p.Arg201Cys) was detected in this tissue by Sanger sequencing but not in peripheral blood leukocytes by both Sanger sequencing and MEMO-PCR (Fig. 2). The conventional Sanger sequencing method from peripheral blood cells did not detect an activating mutation of *GNAS* in any of the eight patients, as expected.

## Discussion

MAS is characterized by various endocrinopathies, including hyperthyroidism, GH excess, and renal phosphate wasting, as well as peripheral PP, as the tissue distribution of Gsα expression is broad [5]. This study described the various clinical characteristics in 14 Korean patients with MAS. In addition, we applied MEMO-PCR to detect somatic mutations in *GNAS* using blood and investigated the clinical response to letrozole in patients with peripheral PP.

Peripheral PP is the most frequent initial presentation of MAS and is much more common in girls than in boys [2]. In this study, peripheral PP was observed in all female patients but not in any males. Treatment of peripheral PP-associated MAS, including AIs and estrogen receptor antagonists, has been evolving for decades [6–10]. However, an ideal pharmacological treatment of peripheral PP-associated MAS has not been identified. AIs prevent the conversion of androgens to estrogens and, thereby, reduce the serum levels of estrogens [3]. Recent reports of the most potent third-generation AIs, anastrozole and letrozole, yielded mixed results [7, 8]. A pilot study of nine girls treated for 12–36 months with letrozole indicated decreased rates of growth, bone maturation, and vaginal bleeding [7]. However, mean ovarian volumes tended to increase over time, and one patient experienced ovarian torsion. A systemic prospective study of anastrozole for the treatment of peripheral PP in 27 girls with MAS over one year found that it was ineffective in halting vaginal bleeding, attenuating rates of skeletal maturation, and increasing linear growth [8]. Tamoxifen, a selective estrogen receptor modulator, was found to have positive results in a year-long multicenter trial of 25 girls with peripheral PP and MAS [9]. However, uterine volumes were unexpectedly found to increase throughout the study and raised safety concerns given the association of tamoxifen and stromal tumors.

In the subset of girls with frequent vaginal bleeding or progressive forms of PP, pharmacological intervention was applied in order to prevent early epiphyseal fusion and reduce the frequency of vaginal bleeding. In this study, the potent third-generation AI letrozole was used for the treatment of peripheral PP with MAS. Letrozole has demonstrated some short-term success in one study [7], but further investigations are needed. Therefore, we also evaluated the safety and efficacy of letrozole. In five patients who were treated with letrozole for two years, there was a significant decrease in estradiol levels; however, bone age was further advanced in four patients. Uterine size did not show a significant change during therapy. Recently, a prospective study revealed that a pure estrogen receptor blocker, fulvestrant, was effective in decreasing vaginal bleeding and rates of skeletal maturation in 13 girls with MAS over 12 months [10]. Long-term studies comparing available medications are needed. Although the PP in MAS is gonadotropin-independent, secondary activation of the hypothalamic-pituitary-gonadal axis may occur, resulting in concurrent central PP. In this study, two patients (Patients 5 and 11) showed central PP at diagnosis. They had initially presented with facial abnormalities at the ages of 9 years and 7 years 1 month, respectively, and they had been referred to an endocrinologist due to previous histories of vaginal bleeding. In addition, one patient (Patient 8) subsequently developed central PP during the treatment of peripheral PP. Central PP in these children might be caused by extensive sex steroid exposure due to uncontrolled peripheral PP [11].

Thyroid disorder is the second most common endocrinopathy in MAS [12]. One retrospective polycentric study analyzed 36 MAS patients, followed over 20 years; 11 patients (31 %) had functional and/or morphological thyroid dysfunctions [13]. In our study, four patients (4/14, 29 %) showed functional and/or morphological thyroid abnormalities, and two of them (2/14, 14 %) had clinical hyperthyroidism with treatment. For this reason, strict monitoring of thyroid function is recommended every six months in patients with MAS [13].

GH excess affects about 20 % of patients with MAS [14, 15]. The median age of GH excess patients in previous reports was 20 years. In this study, all 14 patients were younger than 20, and only two patients showed acromegaly (diagnosed at the ages of 14 and 17 years, respectively). Since acromegaly with MAS is usually accompanied by craniofacial FD, the diagnosis of acromegaly may be delayed by craniofacial FD, masking the dysmorphic craniofacial effect of acromegaly [16]. Therefore, it is important to perform laboratory screening, such as IGF-I, for GH excess in patients with craniofacial FD.

FD is the most common component of MAS [5], and it usually involves the craniofacial bones [17]. Thirteen patients in our study had polyostotic FD involving the craniofacial bones. Isotopic bone scans are useful not only for detecting the extent of the disease but also for quantifying the skeletal disease burden of FD and predicting functional outcome [17].

The relative prevalence of the café au lait spots in the National Institutes of Health (NIH) cohort of patients with FD/MAS was 66 % (140 patients followed over 24 years) [5]. In this study, café au lait spots were found in 9 of our 14 patients (64 %). The mechanism of skin pigmentation in MAS is the activating mutation of Gsα in affected melanocytes and augmentation of tyrosinase gene expression, which results in melanin overproduction on affected melanocytes by increased cAMP-mediated signal transduction [18].

The diagnosis of MAS usually depends on clinical features, and it is not always straightforward, especially in the absence of the classical triad. Genetic analysis of the affected tissue would likely provide diagnostic confirmation of the clinical suspicion of MAS; however, obtaining affected tissues is invasive. We, therefore, applied a non-invasive genetic test to confirm the diagnosis of MAS. Based on the fact that activating *GNAS* mutations mostly occur in the Arg201 residue in MAS, a method for the selective enrichment of Arg201 *GNAS* mutations using a series of nested PCRs and restriction enzyme digestion was developed [19–23]. We performed a simple, practical enrichment technique, MEMO-PCR, for the detection of somatic mutations in *GNAS*. The concept of this technique is similar to that of peptide nucleic acid (PNA)/locked nucleic acid (LNA)-mediated PCR clamping, but the PNA or LNA is replaced by 3′-modified oligonucleotides, which are much less expensive and are easy to design [24]. In this study, the detection rate of MEMO-PCR from peripheral blood leukocytes was 25 % (2/8). Although the test was performed with a small number of patients, the mutation detection rate of MEMO-PCR was not significantly different from that of the previous PNA/LNA-mediated PCR clamping [22, 23].

There are several limitations to our study. MAS is rare, and a limited number of patients had been treated with letrozole therapy; thus, data from an untreated control group of subjects were not available. For this reason, it is difficult to confirm the therapeutic effects of letrozole on patients with MAS. A greater number of subjects and longer periods of treatment are needed before the safety and effectiveness of estrogen receptor modulators such as tamoxifen and fulvestrant as well as AIs including letrozole can be confirmed.

## Conclusions

This study described the various clinical and endocrine manifestations of 14 patients with MAS in a single center in Korea. In addition, this study first applied MEMO-PCR on patients with MAS to detect low-abundance somatic *GNAS* mutation using peripheral blood. A broad spectrum of endocrine manifestations was found in this study. Multiple endocrinopathies should be monitored in patients with MAS through careful physical examinations with history taking and serial endocrine function tests. In this study, we could not definitively conclude the efficacy of two-year letrozole treatment without any severe adverse effects. Better treatment options for peripheral PP and for improving the quality of life of patients with MAS are needed.

## Abbreviations

AI, aromatase inhibitor; CT, computed tomography; FD, fibrous dysplasia; FSH, follicle-stimulating hormone; FT4, free thyroxine; GH, growth hormone; GnRH, gonadotropin-releasing hormone; Gsα, g-protein alpha subunit; LH, luteinizing hormone; MAS, McCune–Albright syndrome; MEMO, mutant enrichment with 3′-modified oligonucleotides; PCR, polymerase chain reaction; PP, precocious puberty; SD, standard deviation; SDS, standard deviation score; T3, total triiodothyronine; TSH, thyroid-stimulating hormone
